# Psychiatry Resident Doctors Experience with Support Following Violence and Aggression Incidents

**DOI:** 10.1192/bjo.2026.11498

**Published:** 2026-06-30

**Authors:** Heba Tariq, Sheikh Aslam, Suresh Thapaliya, Koravangattu Valsraj

**Affiliations:** KMMH, Canterbury, United Kingdom

## Abstract

**Aims:**

Multiple projects across the country are fortunately looking closely at violence and aggression happening to health and social care workers. This is a piece of work around experience of support made available to resident doctors in psychiatry following violence and aggression incidents.

This QI project aims to improve the quality of support and spread awareness about available channels to resident doctors following abuse incidents. A baseline survey was conducted to explore awareness of available channels, rate satisfaction with them, improve them as well as suggest new possible ways of providing support.

**Methods:**

As a part of the QI project, we did a baseline survey to explore whether resident doctors had access to support after a violence and aggression incident and how satisfied they were with it. We then did a root cause analysis (RCA) workshop where resident doctors identified reasons for not accessing these channels.

**Results:**

41 resident doctors participated in the survey. 12 residents reported experiencing anincident of violence and aggression (1–2) times and 8 of these residents were in a junior entry level (CT1–CT3). 22 of these incidents were identified as verbal. 85.71% of residents were not aware of available channels of support in the trust. This coincided with 75% of residents not seeking support. Of the participants that did seek support, 2.44% graded the level of satisfaction with the type of support as 1 (very unsatisfied) and the follow-up answer was (due to lack of communication following the report and not knowing what the outcome of the report was). We then carried out an RCA and the resident doctors identified multiple causes to not seeking support including (not knowing how to access support, feeling speaking up is a burden, feeling the process is not followed through, feeling like they don’t belong due to rotating every 6 months) etc.

**Conclusion:**

The residents have overall agreed there is room for improvement on the level of support available. We used the suggested themes in the RCA to come up with an action plan. Measures we have come up with are including all information around support channels in the induction file given to all new starting resident doctors, spreading awareness about available support on the trust intranet, liaising with the incident reporting system representatives in the trust to improve reporting process and include proper follow up. We are also still meeting regularly to discuss and improve our action plan.

